# Koreans Invested in Making Caregivers Health Important (KIMCHI): A National Community‐based Project to Enhance Knowledge, Attitudes, and Behaviors about Dementia for the Korean American Community

**DOI:** 10.1002/alz.089810

**Published:** 2025-01-09

**Authors:** Bora Nam, Daren Huang, Stacy W Yun, Hye‐Won Shin, Eun Jeong Lee, Nicole Phan, Van Ta Park

**Affiliations:** ^1^ University of California San Francisco School of Nursing, San Francisco, CA USA; ^2^ VA Palo Alto Health Care System, Palo Alto, CA USA; ^3^ Somang Society, Cypress, CA USA; ^4^ AARIN, New York, NY USA

## Abstract

**Background:**

The Korean American older adult population is rapidly growing, with most being foreign‐born individuals with limited English proficiency, and who experience significant health disparities. The Koreans Invested in Making Caregivers Health Important (KIMCHI) project is a national, culturally tailored, bilingual (English/Korean) initiative focused on community‐based research dissemination to empower Korean Americans with insights into healthy cognitive aging and dementia caregiving.

**Method:**

From the community partnership with Somang Society and AARIN, a total of 211 participants, predominantly older adults (aged 24‐ 90 years; M = 69; SD = 12.1), mostly female (70.1%), and foreign‐born (96.2%) with limited English proficiency (61.6%), engaged in the project. Paired‐sample t‐tests were employed to assess mean (M) changes in participants’ knowledge, attitudes, and behaviors regarding Alzheimer’s disease and related dementias (ADRD) pre‐ and post‐test (before and after participating in the KIMCHI presentations).

**Result:**

From the community partnership with Somang Society and AARIN, a total of 211 participants, Significant improvements were observed in post‐test ADRD knowledge (M = 11.51), attitudes (M = 7.13), and behaviors (M = 8.88) compared to pre‐test scores (M = 10.34, M = 6.33, M = 8.11, respectively), t(210) = 1.17, p<.05, t(210) = 0.8, p<.05, and t(210) = 0.76, p<.05, respectively. Moreover, 93.8% of participants expressed satisfaction about KIMCHI, 89.4% learned something new about ADRD, 93.8% found the presentations culturally relevant and applicable, and 89% expressed interest to learn more about ADRD training.redominantly older adults (aged 24‐ 90 years; M = 69; SD = 12.1), mostly female (70.1%), and foreign‐born (96.2%) with limited English proficiency (61.6%), engaged in the project. Paired‐sample t‐tests were employed to assess mean (M) changes in participants’ knowledge, attitudes, and behaviors regarding Alzheimer’s disease and related dementias (ADRD) pre‐ and post‐test (before and after participating in the KIMCHI presentations).

**Conclusion:**

Findings indicate that a community‐based research dissemination project (i.e., KIMCHI) can positively influence ADRD knowledge, attitudes, behaviors, and satisfaction among Korean Americans. Future studies should explore additional culturally tailored interventions, including real‐life case examples, to enhance health‐related outcomes and ensure greater representation in research studies for the Korean American community.

